# Technology-based cognitive training and rehabilitation interventions for individuals with mild cognitive impairment: a systematic review

**DOI:** 10.1186/s12877-018-0893-1

**Published:** 2018-09-15

**Authors:** Shaoqing Ge, Zheng Zhu, Bei Wu, Eleanor S. McConnell

**Affiliations:** 1 Duke University School of Nursing, 307 Trent Drive, Durham, NC USA; 2 Fudan University School of Nursing, Shanghai, China; 3 Fudan University Center for Evidence-Based Nursing, a Joanna Briggs Institute Center of Excellence, Shanghai, China; 4 New York University Rory Meyers College of Nursing, New York, NY USA; 5 Hartford Institute for Geriatric Nursing, New York University, New York, NY USA; 6 Geriatric Research, Education and Clinical Center (GRECC) of the Department of Veterans Affairs Medical Center, Durham, NC USA

**Keywords:** Technology, Cognition, Cognitive training, Cognitive rehabilitation, Systematic review

## Abstract

**Background:**

Individuals with mild cognitive impairment (MCI) are at heightened risk of developing dementia. Rapid advances in computing technology have enabled researchers to conduct cognitive training and rehabilitation interventions with the assistance of technology. This systematic review aims to evaluate the effects of technology-based cognitive training or rehabilitation interventions to improve cognitive function among individuals with MCI.

**Methods:**

We conducted a systematic review using the following criteria: individuals with MCI, empirical studies, and evaluated a technology-based cognitive training or rehabilitation intervention. Twenty-six articles met the criteria.

**Results:**

Studies were characterized by considerable variation in study design, intervention content, and technologies applied. The major types of technologies applied included computerized software, tablets, gaming consoles, and virtual reality. Use of technology to adjust the difficulties of tasks based on participants’ performance was an important feature. Technology-based cognitive training and rehabilitation interventions had significant effect on global cognitive function in 8 out of 22 studies; 8 out of 18 studies found positive effects on attention, 9 out of 16 studies on executive function, and 16 out of 19 studies on memory. Some cognitive interventions improved non-cognitive symptoms such as anxiety, depression, and ADLs.

**Conclusion:**

Technology-based cognitive training and rehabilitation interventions show promise, but the findings were inconsistent due to the variations in study design. Future studies should consider using more consistent methodologies. Appropriate control groups should be designed to understand the additional benefits of cognitive training and rehabilitation delivered with the assistance of technology.

**Electronic supplementary material:**

The online version of this article (10.1186/s12877-018-0893-1) contains supplementary material, which is available to authorized users.

## Background

Due to the aging of the world’s population, the number of people who live with dementia is projected to triple to 131 million by the year 2050 [1, 2]. Development of preventative strategies for individuals at higher risk of developing dementia is an international priority [3, 4]. Mild cognitive impairment (MCI) is regarded as an intermediate stage between normal cognition and dementia [5, 6]. Individuals with MCI usually suffer with significant cognitive complaints, yet do not exhibit the functional impairments required for a diagnosis of dementia. These people typically have a faster rate of progression to dementia than those without MCI [5], but the cognitive decline among MCI subjects has the potential of being improved [7, 8]. Previous systematic reviews of cognitive intervention studies, both cognitive training and cognitive rehabilitation, have demonstrated promising effects on improving cognitive function among subjects with MCI [3, 7, 9, 10].

Recently, rapid advances in computing technology have enabled researchers to conduct cognitive training and rehabilitation interventions with the assistance of technology. A variety of technologies, including virtual reality (VR), interactive video gaming, and mobile technology, have been used to implement cognitive training and rehabilitation programs. Potential advantages to using technology-based interventions include enhanced accessibility and cost-effectiveness, providing a user experience that is immersive and comprehensive, as well as providing adaptive responses based on individual performance. Many computerized cognitive intervention programs are easily accessed through a computer or tablet, and the technology can objectively collect data during the intervention to provide real-time feedback to participants or therapists. Importantly, interventions delivered using technology have shown better effects compared to traditional cognitive training and rehabilitation programs in improving cognitive function and quality of life [11–13]. The reasons for this superiority are not well-understood but could be related to the usability and motivational factors related to the real-time interaction and feedback received from the training system [13].

Three recent reviews of cognitive training and rehabilitation for use with individuals with MCI and dementia suggest that technology holds promise to improve both cognitive and non-cognitive outcomes [14–16]. The reviews conducted by Coyle, et al. [15] and Chandler, et al. [14] were limited by accessing articles from only two databases, and did not comprehensively cover available technologies. Hill, et al. [16] limited their review to papers published until July 2016 and included only older adults aged 60 and above. More technology-based intervention studies have been conducted since then, and only including studies with older adults 60 and above could limit the scope of the review given that adults can develop early-onset MCI in their 40s [17]. Therefore, the purpose of this review is to 1) capture more studies using technology-based cognitive interventions by conducting a more comprehensive search using additional databases 2) understand the effect of technology-based cognitive interventions on improving abilities among individuals with MCI; and 3) examine the effects of multimodal technology-based interventions and their potential superiority compared to single component interventions.

## Methods

### Search strategy

PRISMA guidelines were followed for conducting this systematic review [18]. Based on the research aims and key words, an experienced librarian searched five databases: PubMed (Medline), PsychoINFO (EBSCO*host*), CINAHL (EBSCO*host*), Embase, and Cochrane Library (Wiley). The search strategy used a combination of subject headings and key words for these main concepts: technology, MCI, training, and rehabilitation. The full search strategy is available in Additional file 1. The literature search was limited to empirical studies among human subjects. We did not set boundaries on age since MCI can occur among middle aged to older adults. The literature search was completed on December 1, 2017.

### Inclusion and exclusion of publications

Two authors (SG and ESM) independently reviewed the list of articles found in the literature search. Inclusion criteria were: 1) participants were diagnosed with MCI; 2) a technology-based cognitive training or rehabilitation intervention was evaluated; and 3) an empirical study was conducted. Exclusion criteria were: 1) the effect of the intervention on MCI participants could not be extracted from effects among healthy or dementia participants, and 2) the publication was not in English.

Titles were first reviewed for obvious exclusions. Then, for those retained from the first-round title screening, abstracts were screened. A third-round of full-text screening was then conducted. Any uncertainties or discrepancies between the two authors (SG and ESM) were discussed and resolved.

### Quality assessment

The quality of studies identified as relevant was assessed by two independent reviewers (SG and ZZ) using the Joanna Briggs Institute (JBI) critical appraisal checklist for randomized controlled trials (RCT) and JBI checklist for quasi-experimental studies [19]. Any disagreements that arose were resolved through discussion, or with a third reviewer (BW). The studies were generally methodologically sound with some variations in quality across studies (see Additional file 1: Table S2 and S3).

### Data extraction and synthesis

Two reviewers (SG and ZZ) independently extracted information from each article into the Tables 1 and 2. Disagreements on data extraction were resolved by consensus with the assistance of a third author. A meta-analysis of the 26 articles was inappropriate due to the large variability between the study designs, intervention contents, outcomes measured, and population samples across different studies [20, 21]. We selectively calculated effect sizes for a pair of studies that used the same intervention materials [22, 23]. All data syntheses were conducted by using Revman 5.3 [24]. The forest plot is presented in Additional file 1: Figure S1.

**Table 1:** Sample characteristic of included studies

First author	Year of publication	Location	Setting/context	Sample size^a^	Age^b^ (year)	MCI Criteria	Baseline cognitive characteristic^b^
Cipriani, 2006 [29]	2006	Italy	Dayhospital	10(AD) + 10(MCI)	70.6	Not specified	*MMSE:* 28.0
Rozzini, 2007 [40]	2007	Italy	Medical centers	59	63–78	Petersen criteria	*MMSE: IG1:*26.4 *IG2:*26.0 *CG:*26.8
Talassi, 2007 [36]	2007	Italy	Community-dwelling	37(MCI) + 29(AD)	*IG:*76.2 *CG:*76.1	Not specified	*MMSE: IG*:27.5 *CG*:26.9
Barnes, 2009 [22]	2009	US	Medical centers	47	*IG*:74.1 *CG*:74.8	IWG criteria	*BRANS: IG*:85.2 *CG*:87.8
Finn, 2011 [25]	2011	Australia	Medical center	27	*IG*:69.00 *CG*:76.38	IWG criteria	*MMSE: IG*:28.5 *CG*:27.5
Rosen, 2011 [23]	2011	US	Community-dwelling	12	*IG*:70.67 *CG*:78.00	IWG criteria	*MMSE: IG*:29.33 *CG*:27.83
Gagnon, 2012 [43]	2012	Canada	Medical centers	24	*IG*:68.42 *CG*:67.00	Petersen criteria	*MMSE: IG*:27.83 *CG*:28.08
Herrera, 2012 [42]	2012	France	Medical center	22	*IG*:75.09 *CG*:78.18	Petersen criteria	*MMSE: IG*:27.36 *CG*:27.18
Man, 2012 [13]	2012	Hong Kong	Community service setting	44	*IG*:80.30 *CG:*80.28	Petersen criteria	*MMSE: IG*:21.05 *CG*:23.00
Gonzalez-Palau, 2014 [33]	2014	Spain	Community centers	39(HE) + 11(MCI)	74.60	Petersen criteria	*MEC* 35: 29.61
Han, 2014 [30]	2014	Korea	Medical center	10	72.1	IWG criteria	*MMSE*: 26.7
Hughes, 2014 [45]	2014	US	Community setting	20	*IG*:78.5 *CG*:76.2	MYHAT Cognitive Classification	*MMSE: IG*:27.2 *CG*:27.1
Fiatarone Singh, 2014 [26]	2014	Australia	Community-dwelling	100	70.1	Petersen criteria	*ADAS-Cog: IG1*:8.79 *IG2*:8.29 *IG3*:8.02 *CG:*8.09
Manera, 2015 [32]	2015	France	Medical Center and research unit	9(MCI) + 12(AD)	75.8	National Institute on Aging and Alzheimer Association group clinical criteria	*MMSE:* 27.2
Styliadis, 2015 [34]	2015	Greece	Medical facility	70	*IG1*:71.21 *IG2:*70.42 *IG3*:72.71 *CG1*:71.07 *CG2*:67.64	Petersen criteria	*MMSE: IG1*:25.85 *IG2*:26.21 *IG3*:25.14 *CG1*:26.21 *CG2*:25
Barban, 2016 [39]	2016	Italy, Greece, Norway and Spain	Medical centers	114(HE) + 106(MCI) + 81(AD)	*IG*:74.4 *CG:*72.9	Petersen criteria	*MMSE: IG*:27.3 *CG*:28.1
Gooding, 2016 [35]	2016	US	Medical center	74	75.59	Petersen criteria	*mMMSE: IG1*:51.25 *IG2*:50.29 *CG*:50.39
Heyer, 2016 [28]	2016	US	Community-dwelling	68	*IG*:75.1 *CG*:75.2	IWG criteria	*MMSE:* 26
Klados, 2016 [37]	2016	Greece	Not specified	50	*IG*:69.60 *CG*:67.92	Petersen criteria	*MMSE*: *IG*:26.04 *CG*:25.64
Lin, 2016 [44]	2016	US	Community-dwelling	24	*IG*:72.9 *CG*:73.1	Albert criteria	*MoCA: IG*:24.4 *CG*:25.6
Vermeij, 2016 [31]	2016	Netherlands	Community setting	25(HE) + 22(MCI)	68.4	Petersen criteria	*MMSE* > 27.1 Not specified
Bahar-Fuchs, 2017 [27]	2017	Australia	Community-dwelling	9(MCI) + 11(MrNPS) + 25(MCI+MrNPS)	74.8	National Institute on Aging and Alzheimer Association group clinical criteria	*GDS*: 2.9
Delbroek, 2017 [47]	2017	Belgium	Residential care center	20	*IG*:86.9 *CG*:87.5	Not specified	*Moca: IG*:17.5 *CG*:16.3
Hagovská, 2017 [12]	2017	Slovakia	Outpatient psychiatric clinics	60	*IG*:67.8 *CG*:68.2	Albert criteria	*MMSE: IG*:25.6 *CG*:24.9
Mansbach, 2017 [38]	2017	US	Community-dwelling	38	78.08^c^	Petersen criteria	*BCAT*^*c*^: *IG*:38.65 *CG*:35.72
Savulich, 2017 [41]	2017	UK	Research and medical center	42	*IG*:75.2 *CG*:76.9	Albert criteria	*MMSE: IG*:26.6 *CG*:26.8

*IG* Intervention group, *CG* Control group

^a^*HE* Healthy elderly with no history of neurological or psychiatric deficits, *MrNPS* Mood-related neuropsychiatric symptoms

^b^Data only included elderly with MCI

^c^Demographic data included both MCI and AD

**Table 2:** Overview of included studies

First author and year	Study design	Intervention and Technology	Control	Technology description	Sessions/Duration	Follow-up	Cognitive outcome measures	Other outcome measures	Key findings
Cipriani, 2006 [29]	Pre-post study	Computer based-Cognitive Rehabilitation (cb-CR) programs	NA	TNP software: delivers individualized cognitive rehabilitation exercises in the following cognitive domains: attention, memory, perception, visuospatial cognition, language, and non-verbal intelligence	2 * 16 * 13–45 min sessions for 8 weeks	3 months	MMSE Attention: Visual search; Executive function: Trail Making test A and B; Behavioral Memory: RBMT; Psychomotor learning: digit symbol test; Verbal fluency: phonemic and semantic verbal fluency	Depression (GDS); Anxiety: STAI-X1, STAI-X2; ADL: AADL; QOL: SF-12	MCI: Only significantly improved in memory (RBMT)
Rozzini, 2007 [40]	RCT	TNP + ChEIs	CG1: ChEIs CG2: No treatment	TNP Software	20 * 1 h/session, five days/week for four weeks	1 yr	MMSE Memory: Short story recall; Executive function: Rey’s figure copy and recall, Raven’s colored matrices; Verbal fluency: Letter verbal fluency, Semantic verbal fluency	Mood: depression: GDS; anxiety, apathy Behavioral disturbances: NPI Activities of daily living: BADL	IG: significant improvement in memory, abstract reasoning, and depression CG1: no improvements on any cognition but benefit in depression CG2: no improvement in any outcome measures
Talassi, 2007 [36]	CCT	TNP + OT + BT	PR + OT + BT	TNP Software	30–45 min/session, 4 days/week for 3 weeks	Interven-tion end	MMSE Working memory: forward and backward digit span; Executive function: Rey’s figure copy; Verbal fluency: phonemic and semantic verbal fluency; CDT; Episodic Memory: episodic memory subset of RBMT; Verbal fluency: Phonemic and semantic verbal fluency; Attention: visual search, processing speed: digit symbol test	Mood: depression GDS; anxiety (Stai-Y1,Stai-Y2; ADL: BADL, IADL, PPT;	MCI subjects in IG improved in executive function, visuospatial memory, anxiety, depression, and PPT but not IADL MCI subjects in CG: no improvements
Barnes, 2009 [22]	RCT	cb-CT	Passive computer activities	Computer-based cognitive training software developed by Posit Science Corporation (San Francisco, CA), involving seven exercises including primary and working auditory memory tasks to improve processing speed and accuracy in the auditory cortex	IG: 100 min/day five days/week for 6 weeks CG: 90 min/day, 5 days/week for 6 weeks	Interven-tion end	Global cognitive function: RBANS total score, 5 RBANS index score Memory: CVLT-II Language: COWAT, BNT Executive function: California Trail Making Test Attention: Design Fluency test; Working memory: Spatial Span test	Mood: depression (GDS)	IG showed greater improvement on RBANS total scores but no significant between group difference. Effect sizes for verbal learning and memory measures tended to favor IG. Effect sizes for language and visuospatial function measures tended to favor CG (control group).
Finn, 2011 [25]	RCT	Computerized Cognitive Training Package	No intervention	Lumosity software on a computer contains four or five cognitive exercise that targeted four cognitive domains	30 sessions, 4–5 sessions/week	Interven-tion end	Executive function: IED; Attention: RVP Subjective memory impairment: MFQ Visual memory: PAL	Mood: Depression Anxiety and Stress Scale	IG had significant improvement in visual attention but not processing speed, visual memory, nor mood
Rosen, 2011 [23]	RCT	cb-CT	listening to audio books, reading online newspapers, and playing a visuospatial oriented computer game	Computer-based cognitive training software developed by Posit Science Corporation	IG: 24 sessions, 100 mins/day, 5 days/week CG:24 sessions, 90 min/day, 5 days/week	Interven-tion end	Global cognitive function: RBANS Neuroimaging: fMRI	Not specified	IG > CG: improvement in verbal memory and left hippocampal activation CG: declined in VM
Gagnon, 2012 [43]	RCT	Computer-based VP	AC: Computer-based FP	Computer-based divided attention dual-task training: VP: performing both tasks concurrently and varying allocation priorities across the series of blocks, feedbacks are provided; FP: perform both tasks concurrently and to allocate 50/50 attentional resources to each task, no feedbacks provided	6 * 1 h/session, 3 times/week for two weeks	Interven-tion end	Attention: dual task (digit span task, visual detection task); Executive subtest of TEA Attention: Trail Making Test A; Executive function: Trail Making Test B;	QOL: Well-Being Scale Divided attention: Divided Attention Questionnaire	VP showed significant advantage over FP in improving accuracy and reaction time FP and VP both produced improvements on focused attention, speed of processing, and switching abilities No reliable advantage for VP over FP
Herrera, 2012 [42]	RCT	Computer –based Memory and attention training	Stimulating Cognitive activities	Computer-based cognitive training that involved a memory task and an attention task	24 * 60 min/session twice/week for 12 weeks.	6 months	MMSE-recall; Memory: the forward and backward digit span test, BEM-144 12-word-list recall, the 16-item free and cued reminding test, subscore recall of the MMSE, visual recognition subtest from the Doors and People memory battery, the DMS48 test; executive function: Rey–Osterrieth Complex Figure recall test	N/A	Significant improvement in memory, both episodic recall and recognition
Man, 2012 [13]	CCT	VR-based memory training program	Therapist-led program	VR: participants use either the joystick or the direction buttons of the keyboard to control the navigation action and give responses to a memory task	10 sessions, 30 min/session 2–3 times/week	Interven-tion end	Memory: MMQ; Episodic Memory: FOME	ADL: Lawton IADL	VR: significant improvement in total encoding, total recall, delayed recall and MMQ-strategy Therapist-led: significant improvement in total recall, delayed recall and MMQ-contentment VR > therapist in improving objective memory; Therapist > VR in subjective memory
Gonzalez-Palau, 2014 [33]	Pre-post study	LLM included CTC and PTC	NA	CTC: Gradior Software: a multi-domain cognitive training program including attention, perception, episodic memory and working memory tasks. Principles of feedback and difficulty adaptation are used PTC: FFA: an innovative, low-cost game platform. Work our intensity gradually increases	CTC: 40/session, three times/week for 12 weeks PTC: one-hour session of FFA, three times/week for 12 weeks	Interven-tion end	Global cognitive function: The Mini Examen Cognitivo (MEC 35) Memory: Logical Memory subtests of the WMS III Attention: The Color Trail Test 1 and 2 Verbal learning and memory: HVLT-R	Mood: depression(GDS)	For MCI subjects: Significant improvement in global cognitive function, verbal memory,episodic memory, and decrease in symptoms of depression.
Han, 2014 [30]	Pre-post study	Ubiquitous Spaced Retrieval-based Memory Advancement and Rehabilitation Training USMART Program	NA	USMART program app on IPad	24 face-to-face sessions	Interven-tion end	CERAD-K-N including: verbal fluency: the Categorical Fluency test, the Modified BNT; MMSE; memory: WLMT, WLRT, the Word List Recognition Test, CRT; visuospatial: Constructional Praxis Test; Attention: Trail Making Test A; executive function: Trail Making Test B	N/A	Significantly improved only in memory (WLMT); number of training sessions correlated with WLMT scores
Hughes, 2014 [45]	RCT	Interactive video games (Wii)	Healthy aging education program	Nintendo Wii gaming console for interactive video gaming (bowling, golf, tennis, and baseball)	24 * 90 min, 1 session/week for 24 weeks	1 year	Global cognition: CAMCI; Processing speed/ Attention: Tracking A; Executive function: Tracking B; Subjective cognitive ability	Mood/social functioning: CSRQ-25; ADL: TIADL	IG: No significant improvement in any of the outcome measures. Medium effect size estimates were found for global cognition, subjective cognition, executive function, and gait speed
Fiatarone Singh, 2014 [26]	RCT	IG1: CT + Sham exercise IG2: PRT + Sham cognitive intervention IG3: CT + PRT	Sham exercise + Sham cognitive intervention	COGPACK program: computer-based multimodal and multi-domain exercises targeting memory, executive function, attention, and speed of information processing, including 14 progressively more difficult exercises	CG: 60 min IG: 75 min PRT/CT groups, 100 min combined 2–3 days/week for 6 months	18 months	Global cognition: ADAS-Cog Executive Function: WAIS-III; Verbal fluency: COWAT, animal naming; Memory: BVRT-R, auditory Logical Memory I and II, subsets of WMS-III, List Learning subsection of ADAS-Cog; Attention: SDMT	ADL: B-IADL	CT prevented memory decline only up until 6 months PRT improved global and executive function until 18 months; PRT was better than CT + PRT in improving global and executive function
Manera, 2015 [32]	Pre-post Study	‘Kitchen and Cooking’ Game	NA	Computerized Kitchen and Cooking’ serious game which challenges attention, executive function, and praxis	4 weeks	Interven-tion end	Attention: Trail Making Test A Visual Memory: the Visual Association Test Executive function: the Victoria Stroop Test	ADL: IADL, ADL	Significant improvement in executive function. Improvement in MCI > AD. Longer time played correlated with better executive function
Styliadis, 2015 [34]	CCT	IG1: Long-Lasting Memories (LLM) Intervention: combined cognitive training (CT) and physical training (PT) IG2: CT alone IG3: PT alone	CG1: Active Control (AC) (documentaries viewing) CG2: Passive Control	LLM training system CT and PT as follows: CT: Greek adaptation of Brain Fitness Software: 6 self-paced exercises focused on categories: Attention and Auditory Processing Speed. PT: FFA game platform incorporating Nintendo WII balance games	8 weeks LLM group: Up to 10 h/week PT group: Up to 5 h/week CT group: Up to 5 h/week AC group: Up to 5 h/week	Interven-tion end	Electroencephalogram (EEG) measures of Cortical activity for delta, theta, beta 1 and beta 2 bands	N/A	A significant training effect was identified in the LLM group revealed by EEG measures but no training effects on the MMSE
Barban, 2016 [39]	RCT	Process-based-Cognitive Training (pb-CT) plus reminiscence therapy (RT) + rest	Reminiscence therapy (RT) + pb-CT	SOCIABLE software on a touch screen computer containing 27 games designed to improve function in 7 cognitive domains: attention, executive function, memory, logical reasoning, orientation, language, and constructional Praxis	24 * 1 h treatment sessions, 2 sessions/week for a about 3 months	Interven-tion end	MMSE Memory: RAVLT; Executive function: Rey–Osterreith Complex Figure Test, Phonological Verbal Fluency Test; Executive function: Trail Making Test	IADL	pb-CT: Significant training effects on memory in MCI subjects and the effect was maintained after reminiscence period; Significant training effect on MMSE was not maintained during reminiscence period; Medium effect sizes
Gooding, 2016 [35]	CCT	IG1: Computer based Cognitive Training (cb-CT) IG2: Cognitive Vitality Training (CVT: cb-CT + Neuropsychological and Educational Approach to Remediation (NEAR)	Active Control Group (ACG)	cb-CT: Brain Fitness: repeated drill-and-practice exercises involving memory, attention, and executive functions within domain-specific training modules that allow for adaptive training with titrated difficulty levels. Same CT exercises delivered within a framework that allows for personalization, individual control, and contextualization of exercises	30 * 60 min/session, twice/week for 16 weeks	4 months	Intellectual functioning: WRAT-3; mMMSE; Verbal learning and Memory: the BSRT, the WMS-R LM I and II subtests Visual learning and memory: the WMS-R Visual Reproductions (VR) I and II subtests	Mood: depression (BDI-II)	CVT and cb-CT groups had improvements in global cognition, verbal learning, and verbal memory; CVT and cb-CT had significantly greater improvements than ACG in global cognition, verbal memory, and verbal learning; No significant difference between cb-CT and CVT; Largest mood improvement in CVT, significant difference between CVT and ACG but not between CVT and cb-CT
Hyer, 2016 [28]	RCT	Computerized CT program	Sham cognitive training	Cogmed computer training program: Uses multiple rotating exercises daily that are designed to train working memory.	25 * 40 min /day for over 5 to 7 weeks	3 months	Working Memory: WMS-III Span Board subtest, WAIS-III Letter Number Sequencing subtest; Attention: Trail Making Test Part A; Executive function: Trail Making Test Part B); Subjective memory: CFQ	ADL: the Functional Activities Questionnaire	Significant improvement of executive function, verbal and non-verbal working memory in both CG and IG; Significant improvement of subjective memory in IG but not CG. Significant between group difference in working memory (Span Board) and in adjustment (FAQ)
Klados, 2016 [37]	CCT	Long Lasting Memories (LLM) Intervention (Cognitive Training (CT) + Physical Training (PT))	Active Control (AC): watching documentary and answering questionnaire	Brain Fitness Software FitForAll	CT: 1 h/day, 3–5 days/week for 8 weeks PT: 1 h/day, 3–5 sessions/week for 8 weeks for 8 weeks	Interven-tion end	Cortical Activity, Cortical Functional Connectivity: beta band	Not specified	IG showed beta band functional connectivity of MCI patients
Lin, 2016 [44]	RCT	VSOP	MLA	Software INSIGHT: online program designed by Posit Science, included five training tasks: eye for detail, peripheral challenge, visual sweeps, double decision, and target tracker	1 h/day 4 days/week for 6 weeks	Interven-tion end	Processing speed: The Useful Field of View Executive function: The EXAMINER	ADL: TIADL Neuroimaging data: magnetic resonance imaging	IG > CG: improvement in trained (processing speed and attention) and untrained (working memory) cognitive domains, IADL, CEN and DMN
Vermeij, 2016 [31]	Pre-post study	WM training program	NA	Cogmed computer program	25 sessions, 45 min per session for 5 weeks	3 months	Working memory: WAIS-III Digit Span forward and backward, WMS-III Spatial Span forward and backward; Verbal memory: Dutch equivalent of RAVLT; Figural Fluency: RFFT; Cognitive impairment: CFQ	N/A	IG: Significant improvement in trained verbal and visuospatial working memory tasks as well as executive function. Training gain was larger in the healthy elderly (HE) and was only maintained among HEs. Improvements in non-trained near-transfer tasks, maintained after 3 months follow-up
Bahar-Fuchs, 2017 [27]	RCT	home-based individually-tailored and adaptive cb-CT	AC	CogniFit Software: a computer-based program involving 33 tasks designed to train a broad range of cognitive abilities	2 sessions/day, 3 days/week, for 8–12 weeks	12 weeks	Composite score global cognition Memory: L’Hermitte Board, Logical Memory, RAVLT Verbal fluency: SydBat Processing speed: the Trails A and B tasks Self-reported cognitive function	Mood	MCI in IG: greater improvement in memory, learning, and global cognition. No training effect in mood, self-reported memory Training gains in MCI (including ADL) were consolidated over time large effect sizes of intervention at the follow-up assessments in learning, delayed memory, and global cognitive function, medium effect size in non-memory composite
Delbroek, 2017 [47]	RCT	VR dual task training with the BioRescue	No intervention	BioRescue Software: nine exercises to train balance, weight bearing, memory, attention and dual tasking. Led by a therapist, participants stand on a platform, adjustable difficulties based on performances	Gradually increased from 18 min in week 1 to 30 min in week 5	Interven-tion end	The Dutch version of MoCA	Motivation: The Dutch version of IMI emotions: OERS	IG significantly improved on balance, but not on global cognitive function or cognitive-motor dual tasking or gait performance
Hagovská, 2017 [12]	RCT	Cb-CT	Classical group-based cognitive training	CogniPlus program: on a computer, includes five sub-programs that involved activities that are similar to everyday activities, targets attention, working memory, long-term memory, planning of everyday activities, and visual-motor abilities.	20 *30 min, 2 sessions/week for 10 weeks	10 weeks	Self-reported functional activities: FAQ Global cognition: ACE Attention: The Stroop Test	QOL: Spitzer QOL index Functional activities: The Functional Activities Questionnaire	IG demonstrated larger improvements in QoL and attention than CG. The transfer to functional activities was the same between groups
Mansbach, 2017 [38]	CCT	cb-CR	No intervention	Memory Match online cognitive rehabilitation module: designed to improve attention and visual memory, requires the participant to visually pair “matching pictures” by remembering their location	9*30 min/session	Interven-tion end	Global cognitive functioning: BCAT AD8 Dementia Screening Interview, KPT	Attitudes about their cognitive abilities: SRI Mood: depression: GDS-SF	IG > CG in global cognition at post-intervention assessment
Savulich, 2017 [41]	RCT	CT	No intervention	Game Show on iPad: a novel learning and memory game, target to improve episodic memory	8 sessions, 1 h/session	Interven-tion end for 4 weeks	MMSE; Episodic memory and new learning: CANTAB PAL; Visual/spatial abilities: BVMT-R; Processing speed: CANTAB CRT	GDS-SF Anxiety and depression: HADS; Apathy: AES	IG > CG: significantly better performance in episodic memory, visuospatial abilities, MMSE, and less apathy

*Pb-CT* = Process-based cognitive training, *Cb-CR* = computer-based cognitive rehabilitation, *Cb-CT* = computer-based cognitive training, *TNP* = Neuropsychological Training, *WLMT* = memory Word List Memory Test, *USMART* = Ubiquitous Spaced Retrieval-based Memory Advancement and Rehabilitation Training, *MSS* = Memory Support System, *PT* = Physical training, *CT* = cognitive training, *LLM* = long lasting memories, *CCT* = clinical controlled trials, or, computerized cognitive training, *WM* = working memory, *ACG* = Active Control Group, *CVT* = Cognitive Vitality Training, *NEAR*: motivational therapeutic milieu based on Neuropsychological and Educational Approach to Remediation (NEAR) model, *FFA* = FitForAll, *PRT* = Progress resistance training, *VR* = virtual reality, *PR* = Physical rehabilitation, *OT* = Occupational therapy, *BT* = Behavioral training, *ChEIs* = cholinesterase inhibitors, *VP* = Variable Priority, *FP* = Fixed Priority, *VSOP* = Vision-based speed-of-processing, *MLA* = mental leisure activities, *TIADL* = The Timed Instrumental Activities of Daily Living, *QOL-AD* = The Quality of Life-AD, *PTC* = physical training component, *CAMCI* = The Computerized Assessment of Mild Cognitive Impairment, *CSRQ-25* = Cognitive Self-Report Questionnaire-25, *RAVLT* = the Rey Auditory Verbal Learning Test, *AADL* = advanced activity of daily living, *RBMT* = Rivermead behavioral memory test, *RBMT* = Rivermead behavioral memory test, *CERAD-K-N* the Korean version of the CERAD Neuropsychological Assessment Battery, *DRS-2* = Dementia Rating Scale-2, *E-Cog* = The Everyday Cognition, *WAIS-III* = Wechsler Adult Intelligence Scale-Third Edition, *WMS-III* = Wechsler Memory Scale-Third Edition, *CFQ* = Cognitive Failures Questionnaire, *RFFT* = the Ruff Figural Fluency Test, *SCWT* the Stroop Color-Word Task, *IADL* = Instrumental Activities of Daily Living scale, *ADL* = the Independence in Activity of Daily Living index, *WRAT-3* = Wide Range Achievement Test–3rd Edition, *BSRT* = Buschke Selective Reminding Test, *MFQ* = Memory Functioning Questionnaire, *IED* = Intra−/extra-dimensional set shifting, *RVP* = a mea Rapid visual information processing, *MFQ* = Memory Functioning Questionnaire, *PAL* Paired-associates learning, *SDMT* = Symbol Digit Modalities Test, *BVRT-R* = Benton Visual Retention Test-Revised 5th Edition, *B-IADL* Bayer Activities of Daily Living, *ADAS-Cog* = Alzheimer’s Disease Assessment Scale-cognitive subscale, *MoCA* = the Montreal Cognitive Assessment, *IMI* = Intrinsic Motivation Inventory, *OERS* = Observed Emotion Rating Scale, *NPI* = Neuropsychiatric Inventory, *BADL* = Basic Activities Daily Living, CDT = clock-drawing test, *PPT* = physical performance test, *TEA* = Test of Everyday Attention, *GDS* = Geriatric Depression Scale, *GAI* = Geriatric Anxiety Scale, *AES* = Apathy Evaluation Scale, *MMQ* Multifactorial Memory Questionnaire, *FOME* = Fuld Object Memory Evaluation, *CVLT-II* = California Verbal Learning Test – II, *COWAT* = Controlled Oral Word Association Test, *BNT* = Boston Naming Test, *BCAT* = The Brief Cognitive Assessment Tool, *FAQ* = Functional Activities Questionnaire, *ACE* = Addenbrooke’s Cognitive Examination, *EXAMINER* = Executive Abilities: Measures and Instruments for Neurobehavioral Evaluation and Research, *BCAT* = The Brief Cognitive Assessment Tool, *SRI* = self-rating inventory of Cognitive Ability, *KPT* = Kitchen Picture Test of Practical Judgment, *GDS-SF* = Geriatric Depression Scale-Short Form, *CANTAB PAL* = Cambridge Neuropsychological Test Automated Battery Paired Associates Learning, *BVMT-R* = Brief Visuospatial Memory Test-Revised, *CANTAB CRT* = Cambridge Neuropsychological Test Automated Battery Choice Reaction Time, *AES* = Apathy Evaluation Scale, *HADS* = Hospital Anxiety and Depression Scale, *CEN* = central executive network, *WLRT* = Word List Recall Test, *MN* = mode network, *CRT* = Constructional Recall Test, *VTA* = Visual Association Test

### Results

**Fig. 1:**
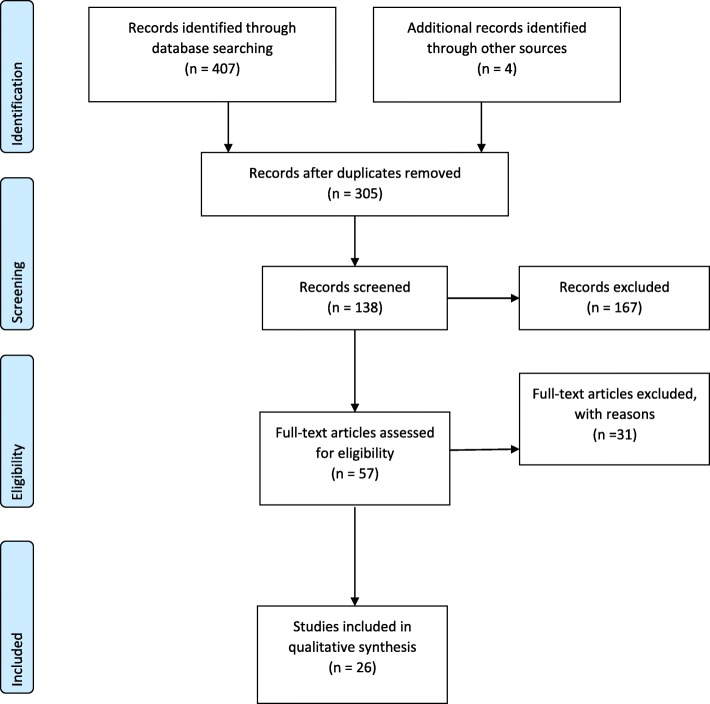
PRISMA Flow Chart

Based on the strategy and criteria described above, 26 of 411 studies identified were deemed eligible for review. The PRISMA flowchart in Fig. 1 presents the decision pathway for final inclusion of studies.

### Quality of the studies

Additional file 1: Tables S2 and S3 summarize the quality assessment of the 26 eligible studies using the JBI criteria, which included both randomized controlled trials and quasi-experimental studies. Of these, 15 were randomized controlled trials (Table 2), 6 were controlled clinical trials, 5 were pretest-posttest studies (Table 2).

Among the RCT studies, only four articles [12, 25–27] reported the procedure for randomization. Two studies allocated the participants by utilizing computer-generated random numbers [26, 27], while the other two studies [12, 25] randomized participants by having an independent person use sealed envelopes. The remaining 11 articles did not report the randomization procedure used for allocating participants. Only three studies [26–28] reported that they were double-blinded sham-control trials.

In the quasi-experimental studies, five studies [29–33] utilized pretest-posttest design. The convenience samples and limited sample sizes (*n* = 10; *n* = 10; *n* = 22; *n* = 9; *n* = 11) restricted their generalizability. Six studies utilized a controlled clinical trial design [13, 34–38]. All the studies lacked external validity due to use of convenience samples or sampling methods that were not clearly described. Quasi-experimental studies which lacked randomization also were limited by a potential allocation bias.

### Sample characteristics of the included studies

The sample and design characteristics of each study are summarized in Table 1. More than 40% of the included studies were published from 2016 to 2017 (*n* = 11). Studies were conducted in different countries: United States (*n* = 7), Italy (*n* = 3), Australia (*n* = 3), France (*n* = 2), Greece (*n* = 2), Canada (*n* = 1), Hong Kong (*n* = 1), United Kingdom (n = 1), Belgium (*n* = 1), Slavonia (*n* = 1), Spain (*n* = 1), South Korea (*n* = 1), and Netherlands (*n* = 1). Only one study [39] reported recruitment across multiple countries.

The total number of the participants with MCI included in this systematic review was 1040. Seven studies included both participants living with MCI and those with other cognitive statuses (either individuals who were cognitively normal or individuals who had dementia), and reported intervention effects for groups of MCI individuals. The mean age ranged from 67.8 and 87.5. All but three studies reported the criteria used to diagnose MCI. Peterson criteria were used in 12 studies, International Working Group (IWG) criteria were used in five studies, Albert criteria were used in three studies, National Institute on Aging and Alzheimer Association workgroup clinical criteria were used in two studies, and Monongahela-Youghiogheny Healthy Aging Team (MYHAT) Cognitive Classification criteria were used in one study.

### Characteristics of interventions

#### Single-component interventions

The majority of the studies (*n* = 18, or 69%) involved single-component technology-based cognitive interventions (Table 2). Characteristics of the interventions varied widely. Seventeen different interventions were utilized in the cognitive training programs studied (see details in Table 2).

#### Multimodal interventions

Eight studies utilized multimodal interventions (Table 2). One approach was cognitive training combined with different types of therapies [35, 36, 39, 40]; another approach was cognitive training combined with physical training [26, 33, 34, 37].

##### Cognitive training plus therapy

Four studies combined the technology-based cognitive training with other therapies as intervention, specifically reminiscence therapy [39], Neuropsychological and Educational Approach to Remediation (NEAR) [35], occupational therapy [36], and medications (cholinesterase inhibitors (ChEIs)) [40]. Two types of software were involved in the cognitive intervention component, including SOCIABLE [39], and NeuroPsychological Training (TNP) [36, 40]. The training sessions lasted for a minimum of 3 weeks [36] to a maximum of 16 weeks [35].

##### Cognitive training combined with physical training

Four studies examined the combined effects of technology-based cognitive and physical training. Two studies used the Long-Lasting Memories (LLM) intervention to provide integrated cognitive and physical training [34, 37], and the physical component was delivered using the FitForAll platform. Gonzalez-Palau, et al. [33] also used the FitForAll platform to provide physical training, but used Gradior program to deliver the cognitive training. Singh and colleagues used Pneumatic resistance machines (Keiser Sports Health Equipment, Ltd) to provide progressive resistance training [26]. The length of these physical trainings lasted from 6 weeks [34] to 6 months [26].

### Overview of technologies

The studies reviewed used the following types of technologies: traditional keyboard computers (*n* = 16), touch screen computers (*n* = 4), gaming consoles or platforms (*n* = 5), and tablets (*n* = 3). Gonzalez-Palau, et al. [33] and Styliadis [34] used both computer and gaming platforms in their interventions. Since 2014, technologies that are more interactive and immersive (virtual reality, gaming console, exergaming platform) have been introduced in cognitive intervention studies.

Compared to traditional therapist-led or pen and paper cognitive interventions, technologies are “smarter” in tracking participants’ performances and adjusting the intervention difficulty. By applying technologies as a delivery method, researchers were able to record the participants’ performance throughout the intervention process. Thirteen studies tracked participants’ performance as one of the outcome variables. Twelve studies used intervention programs that could adjust the intervention difficulties to keep challenging the participants’ abilities, as well as avoid distressing them with too many training failures.

### Effects of interventions

#### Cognitive outcomes

##### Global cognitive function

Twenty-two studies assessed the effects of the interventions on global cognitive function (Table 2). Various instruments were used, including MMSE, Repeatable Battery for Assessment of Neuropsychological Status (RBANS), Computerized Assessment of Mild Cognitive Impairment (CAMCI), Alzheimer’s Disease Assessment scale-cognitive subscale (ADAS-Cog), Brief Cognitive Assessment Tool (BCAT), Addenbrooke’s Cognitive Examination (ACE), Montreal Cognitive Assessment (MoCA), Spanish version MMSE (MEC35), and composite score from measured cognitive domains.

Out of the 22 studies, eight studies found their intervention significantly improved global cognitive function among individuals with MCI. These studies used different cognitive interventions, all but one [20] of them were interventions targeting multiple cognitive domains. Five studies used an active control group, and three of them found a significant between-group difference in global cognition improvement. Barban, et al. [39] reported a significant treatment effect of a computerized multi-domain process-based cognitive training combined with reminiscence therapy in MMSE mean scores (Cohen’s *d* = 0.44). Gonzalez-Palau, et al. [33] reported a significant improvement in global cognitive function (MEC35) among MCI individuals who went through a multi-domain cognitive training program including both cognitive and physical training components. Gooding, et al. [35] compared the computerized cognitive training and cognitive vitality training to an active control group, and reported significantly larger improvements in both intervention groups than the active control group in mMMSE mean score [*F* (2, 71) = 11.56, *p* < 0.001, ${\eta}_p^2=$ 0.25] with a medium effect size (Cohen’s *d* = 0.30 – 0.53). However, this training effect was not maintained at 3-month follow-up. Bahar-Fuchs, et al. [27] reported a significantly greater improvement in global cognition composite score in the training group than the active control with a large effect size (Cohen’s *d* = 0.80). On the other hand, Barnes, et al. [22] found significant RBANS total score improvement in the intervention group after an auditory processing speed and accuracy training, but the between-group difference compared to the active controls was not significant (SD = 0.33). All the other three studies that did not use an active control found significant between-group differences in changes in global cognitive function [26, 38, 41].

Two studies that compared the computer-based cognitive training with listening to audio books, reading online newspapers, and playing a visuospatially-oriented computer game met the requirement for meta-analysis [22, 23]. The design of study, content of intervention, duration and length of follow-up were similar. A total of 59 individuals were included in the meta-analysis. In Additional file 1: Figure S1, the pool weighted standard mean difference score of RBANS total score was 1.62 (95% CI: -1.63 - 4.87). This result indicated that there was no significant difference in the effectiveness for computer-based cognitive training in improving RBANS total score for individuals with MCI after intervention.

##### Attention and working memory

Eighteen studies assessed the effects of technology-based cognitive training or rehabilitative programs on attention/working memory, which are required for storage of new information. The most commonly used measures were the digit span test. Other measures included the spatial span test, Trail Making Test A and B, visual search, spanboard, dual task (digit span task + visual detection task), subscale of Addenbrooke’s Cognitive Examination, and Symbol Digit Modalities Test (SDMT).

Out of 18 studies, eight studies reported significant improvement in attention/working memory. Two studies compared computerized training programs (Cogmed Software) with no intervention or a sham cognitive intervention [28, 31]. Significant improvements on spanboard (*p* = .01) [28], digit span (*p* < .01) [31], and spatial span (*p* < .05) [31] performance were observed at a 3-month follow-up in the intervention group. Other interventions included memory and attention training, variable priority training, and vision-based speed-of-processing training. Significant improvements were found in digit span forward ability (${\eta}_p^2$= .14, *p* < .05) [42], accuracy (*p* = 0.001), reaction time (*p* < .01) [43], spatial span (*p* = .003) [22] and working memory (${\eta}_p^2$ = .28, *p* = .01) [44]. However, three other studies that measured attention using digit span did not report significant results [23, 33, 36].

In terms of technologies used among the eight studies, all of them applied computerized programs to deliver the interventions. Specifically, they all used a keyboard, not a touch screen, to record the test responses.

##### Executive function

Sixteen studies assessed the effect of technology-based cognitive intervention on executive function. Among them, six studies used the Trail Making Task B, six studies used the phonemic and semantic fluency test, four studies used the Rey-Osterreith Complex Figure Test. Other measures included: WAIS Matrices, Ruff Figural Fluency Test, Test of Everyday Attention (TEA), the intra−/extra-dimensional set shifting, and Executive Abilities: Measures and Instruments for Neurobehavioral Evaluation and Research (EXAMINER).

Out of the 16 studies, nine studies reported significant improvement in executive function [22, 26–28, 31, 32, 36, 40, 44]. The interventions used in these studies included both multi-domain cognitive training, specific training tasks, and gaming. The length of interventions ranged from 3 [36] to 24 weeks [26]. Interestingly, three studies used TNP software as an intervention component [29, 36, 40]; although the intervention length varied, two out of the three studies found significant improvement in executive function but used different measures [36, 40]. Talassi [36] found that TNP integrated with occupational therapy and behavioral therapy had a significant improvement in the Rey-Osterreith Complex Figure Test. Rozzini and Costardi [40] found that MCI individuals receiving cognitive training and ChEIs reported significant improvements in Ravens Coloured Progressive Matrices post-intervention (*p* < 0.02). This beneficial effect was not found when using TNP only [29]. However, both Talassi [36] and Rozzini [40] failed to report an effect size for their intervention effect. Other studies that demonstrated significant improvements in executive function varied greatly in terms of sample size, intervention content, total intervention time, and executive function measures.

##### Memory

Nineteen studies assessed memory. Sixteen out of the 19 studies found a significant effect on memory. The measures of memory varied greatly. Major measures included the Wechsler Memory Scale (WMS) and Rey Auditory Verbal Learning Test (RAVLT). Four studies used the WMS-III and three out of the four studies found significant improvements in memory after intervention. The intervention used in the three studies included Cogmed computer program [31], Game show on iPad [41], and LLM including both cognitive and physical training components [33]. The intervention period ranged from 5 to 12 weeks and significant improvement in memory was reserved until end of the three months’ follow-up [31]. The other study used GOPACK multi-domain cognitive training program to conduct a 6-month intervention but did not find a significant improvement in memory measured by subsets of WMS-III [26]. Three studies used RAVLT to measure verbal memory and all of them found significant benefit of the cognitive interventions being used [27, 31, 39]. The interventions included SOCIABLE, Cogmed, and CogniFit software programs, with the intervention lengths ranged from 5 to 12 weeks and follow-up period up to 3 months [27, 31]. Among the three training software programs, Cogmed targeted on working memory, while the other two targeted on multiple cognitive domains.

Other studies that studied memory as an outcome variable each used various measures including the 12-word-list recall test from the BEM-144 memory battery, the 16-item free and cued reminding test, Buschke Selective Reminding Test, Hopkins Verbal Learning Test, Auditory Logical Memory, Short story recall, WMS-R Visual Reproductions I and II subtests, Pattern recognition memory, Benton Visual Retention Test, short story recall, Rivermead behavioral memory test, and Brief Visuospatial Memory Test-Revised. The interventions of these studies lasted for 3 [36] to 16 weeks [35] with the follow-up time up to 6 months. All but one [32] of these studies demonstrated significant improvements in memory. Manera et al. [32] found the 4-week “kitchen and cooking game” intervention had no significant effect on improving memory.

In terms of technologies, all but two studies used computer to deliver the interventions and used keyboard to collect the data. Only two studies used iPad [41] and VR technology [38] to deliver their memory training programs.

#### Non-cognitive outcomes

##### Mood

Nine studies assessed depression. The most commonly used measures were Geriatric Depression Scale (GDS) and its short form GDS-SF [22, 29, 33, 36, 38, 40, 41], other inventory used included Beck’s Depression Inventory [35] and Depression Anxiety and Stress Scale [25]. Four studies reported significant reduction of depression among individuals with MCI [33, 35, 36, 40]. None of these studies reported effect sizes for their interventions reducing depression. Two out of these four studies used a multimodal intervention that also integrated physical trainings [33, 36].

Five studies assessed anxiety [25, 29, 36, 40, 41]. The most often used measure was the State Trait Anxiety Inventory (STAI) [29, 36] used in two studies. Only one study showed a significant reduction in anxiety for individuals with MCI. Talassi used a multimodal intervention including cognition, behavioral, and occupational training compared to its control group that had physical rehabilitation, occupational, and behavioral training, and found that the intervention group had significant decrease in anxiety but not the control group participants [36].

##### ADL

Eleven studies assessed ADL as a secondary outcome. The Basic Advanced and Instrumental Activities of Daily Living scales were used in 5 studies [29, 36, 40, 44, 45]. Other measures included the Functional Activities Questionnaire [28], B-IADL scale [26], and HK Lawton IADL [13]. Two out of nine studies reported a statistically significant improvement in ADL [27, 44]. However, only one study found a significant between-group difference with a small to medium effect size (η^2^ = 0.21) [44].

##### Quality of life

Three studies assessed quality of life [12, 29, 43]. Measures included SF-12 [29], Well-Being Scale [43] and Spitzer-QOL [12]. Only one study reported significant results. Hagovská, et al. [12] found that technology-based cognitive training produced a larger improvement in QoL than classical group-based cognitive training with a medium effect size (*r* = 0.69).

## Discussion

In the past decade, technology-based cognitive interventions have gained increased research interest worldwide. Almost half (42%) of the studies reviewed were published in 2016 and 2017, suggesting the growth in the importance of technology-based interventions. The vast majority of the studies were conducted in developed countries, which may be associated with the limited availability of and familiarity with technology among older adults in lower income countries.

The types of technology used varied across studies and included computers, tablets, VR, and gaming consoles. Computers were the most widely used technology with 77% using computers to assist delivery of cognitive interventions. The majority utilized commercially available software or programs, with only nine of the studies used training programs developed by the study researchers for the specific study purpose. Therapists or coaches were used to teach, assist, or even supervise the use of technologies along the intervention process. In nearly half of studies (*n* = 12) therapists provided instructions at the beginning of the intervention, or provided help throughout the intervention. All but two studies were conducted using only one type of technology, so no conclusions can be drawn regarding the effect of different types of technology on intervention results. Comparing effects of technology across studies was not possible due to the variability among interventions. With the rapid development of technologies, we can anticipate new types of technologies being utilized to assist cognitive training and rehabilitation interventions in the future.

Overall, technology-based cognitive training and rehabilitation have demonstrated promising beneficial effects on various domains of cognition with moderate to large effect sizes. Most studies (e.g., [28, 31, 44]) assessed participants on different cognitive domains that were not limited to the trained task, but also in other non-trained tasks and other cognitive domains, suggesting a transferable beneficial effect of cognitive training and rehabilitation. For example, Hyer, et al. [28] found that working memory training also improved executive function among trained MCI participants, and the impact was preserved until the end of the 3-month follow-up. This transferability is consistent with previous systematic reviews [14, 15]. However, the training gain and transferability of the training gain varied by intervention (e.g., [22]) and delivery method (e.g., [13]). Therefore, future studies are still needed to understand which intervention would benefit various cognitive domains most efficiently.

Only one study included in this review examined whether applying technology as the delivery method would have a stronger effect on the intervention outcomes, in comparison to the use of a traditional manual delivery. Man, et al. [13] compared the training effect of a memory training program delivered with a non-immersive VR-based system versus with color-print images that matched the VR images. This study found that the VR group showed greater improvement in objective memory but the non-VR group reported greater contentment with memory performance, highlighting the potential importance of receiving verbal and emotional support from training therapists on improving participants’ satisfaction with their memory performance. This study shows that depending on the outcomes that an intervention targets, technology-based and manual trainings may have their own strengths and weaknesses. No conclusions can be made whether one type of intervention is generally more effective than the other.

The effect of the same technology-based cognitive intervention seems to vary between groups with different level of cognitive decline. Some but still limited evidence suggested that participants without cognitive impairment seem to obtain a larger cognitive improvement from technology-based cognitive interventions than those with MCI. Vermeij, et al. [31] found that healthy participants had a larger gain in both trained working memory tasks and untrained executive function tasks than those with cognitive impairment. However, the findings are not conclusive. Barban, et al. [39] found that process-based cognitive training improved verbal memory among MCI participants and improved executive function among healthy participants. On the other hand, participants with MCI seem to gain a larger cognitive benefit than those with Alzheimer’s disease (AD). Cipriani, et al. [29] fount that the TNP program significantly improved memory and global cognition among participants with MCI, but only improved memory among those with AD. Similarly, Manera, et al. [32] found that the serious cooking game significantly improved executive function among participants with MCI but not those with AD.

Measures of physical function and mood were used in most studies, but unfortunately most of these were used to ensure non-biased randomization assignment at baseline rather than as outcome measures, so we have limited understanding of the effects of technology-based cognitive interventions on these outcomes. Nine studies evaluated mood (e.g., depression, anxiety) as an outcome, and eleven studies included ADL as an outcome variable. Among these, four studies found technology-based cognitive interventions had beneficial effect on mood and two studies found beneficial effect on functional activity. Technology-based cognitive training studies included in this review may have limited impact on mood and functional activity.

Two studies of technology-based cognitive training and one study of technology-based cognitive rehabilitation examined the effect of their interventions on quality of life [12, 29, 43]. However, only one of the four studies reported significant result. Hagovská, et al. [12] found that technology-based cognitive training produced a larger improvement in QoL than classical group-based cognitive training. This lack of effect of cognitive intervention on QoL is consistent with previous systematic review on the efficacy of cognitive interventions on QoL [14]. However, each study used a different QoL instruments, and various research designs (e.g., types of interventions, lengths of follow-up, and types of control groups). Given the limited number of studies conducted, future studies using comparable designs are still needed to further understand the effectiveness of the intervention on quality of life.

A previous systematic review suggested that multimodal cognitive inventions were a promising research area [15]. In our review, we found eight studies that applied multimodal interventions combining technology-based cognitive training and physical exercise or other therapeutic methods. We expected to see the findings of these studies would provide support for speculation that multimodal cognitive interventions would produce a greater impact on improving cognitive function as well as mood and functional abilities. However, the eight reviewed articles provided insufficient evidence to support this conjecture. Five out of the eight studies were not designed to compare the efficacy of multimodal cognitive intervention compared to cognitive intervention alone [33, 34, 36, 37, 39], and it was difficult to draw any conclusions from the remaining three studies remained due to the great variability in the designs across studies. According to one study, customized technology-based cognitive training produced additional benefit, and technology-based cognitive intervention plus ChEIs was superior to ChEIs alone [35]. Interestingly, Fiatarone-Singh, et al. [26] found that progressive resistance training produced more improvement in executive function and global cognition than both cognitive training and multimodal intervention including cognitive training and progressive resistance training. Findings from this study suggest that physical exercise may particularly benefit executive function, but that implementing multimodal cognitive and physical interventions may be too challenging for people with MCI. Previous systematic review on the efficacy of combined cognitive and exercise intervention in older adults with and without cognitive impairment did not find sufficient evidence to confirm the beneficial effect among older adults with cognitive impairment [46]. Taken together, more studies are needed to understand the advantages of a multimodal cognitive intervention in individuals with MCI. Future studies should design appropriate control groups to understand the additional value produced by a multimodal cognitive intervention than a single model intervention. Additionally, researchers should also bear in mind the possibility that older adults with MCI may not be able to manage the cognitive challenge associated with multimodal interventions.

The studies reviewed generally had small sample sizes, ranged widely from 10 [13] to 301 participants [39]. The average sample size across studies was 54; 39% of the studies had sample sizes of less than 30. The small sample sizes may be related to the complicated diagnostic criteria of MCI, the ethical challenges of conducting intervention studies in older adults with MCI, and the limited availability of some technology-based cognitive intervention programs. More importantly, potential beneficial effects of an intervention could be diminished due to a small sample size.

The measures applied varied greatly across studies, which created challenges in the comparison and generalizability of the study findings. Future studies should consider using measures that have been shown to have good validity and reliability as well as have been frequently used among the MCI population (e.g., CES-D, MMSE, QOL-AD, etc.). Neuroimaging techniques have emerged to be more widely used to obtain information on how technology-based cognitive interventions would affect neural connectivity [37], activation [23], and brain atrophy [31].

We conducted a comprehensive search of literature on the topic area using five major databases. However, this systematic review should be considered in light of its limitations. We only reviewed articles in English language. There may be other relevant studies that were published in other languages. We also need to be aware that technology is developing rapidly so that promising technology-based cognitive training and rehabilitation programs may exist that have not yet been published due to concerns about protecting participants.

## Conclusion

The findings from this systematic review suggest that technology-based cognitive training and rehabilitation programs show promise for improving cognitive function, with some interventions showing moderate to large effect sizes. Computers, tablets, gaming consoles and platforms, and VR systems were the common types of technologies used. Both general and domain-specific cognitive training have led to improved cognition, primarily in memory, but with some evidence that executive function may also be positively affected. Studies that examined the impact of cognitive training on improving mood and functional abilities, have generated less convincing evidence. Multimodal intervention programs integrated technology-based cognitive intervention and other therapies have produced inconsistent findings on the superiority over only applying technology-based single model cognitive intervention. Overall, technology-based cognitive training and rehabilitation are promising intervention methods to improve cognitive function. Future studies should put effort to clarify whether the added benefits of implementing multimodal interventions exist, and carefully consider the potential extra burden caused to individuals with MCI. Additionally, future studies should aim to lessen the variabilities in intervention design and measures applied.

## Additional file

Additional file 1: **Table S1.** Searching Strategy. **Table S2.** Results of quality assessment based on JBI critical appraisal checklist for randomized controlled trials*. **Table S3.** Results of quality assessment based on JBI critical appraisal checklist for quasi-experimental studies*. **Figure S1.** Forest plot - RBANS score (computer-based intervention VS. Control). (DOCX 34 kb)

## Abbreviations

ACE: Addenbrooke’s Cognitive Examination
AD: Alzheimer’s disease
ADAS-Cog: Alzheimer’s Disease Assessment scale-cognitive subscale
BCAT: Brief Cognitive Assessment Tool
CAMCI: Computerized Assessment of Mild Cognitive Impairment
ChEIs: cholinesterase inhibitors
GDS: Geriatric Depression Scale
GDS-SF: Geriatric Depression Scale short form
IWG: International Working Group
JBI: Joanna Briggs Institute
LLM: Long-Lasting Memories
MMSE: Mini-mental state examination
MoCA: Montreal Cognitive Assessment
MYHAT: Monongahela-Youghiogheny Healthy Aging Team
NEAR: Neuropsychological and Educational Approach to Remediation
RAVLT: Rey Auditory Verbal Learning Test
RBANS: Repeatable Battery for Assessment of Neuropsychological Status
RCT: randomized controlled trial
SDMT: Symbol Digit Modalities Test
STAI: State Trait Anxiety Inventory
TEA: Test of Everyday Attention
TNP: Neuropsychological Training
VR: virtual reality
WMS: Wechsler Memory Scale
