# Supplementary material for: Technology-based cognitive training and rehabilitation interventions for individuals with mild cognitive impairment: a systematic review
Source: BMC Geriatr. 2018 Sep 15;18:213. doi: 10.1186/s12877-018-0893-1 (PMC6139138; doi:10.1186/s12877-018-0893-1)

**Additional file**

**Table S1. Searching Strategy**

Date: 12/1/2017

Database: PubMed (MEDLINE)

| Set # |  | Results |
| --- | --- | --- |
| 1 | technology[MeSH] or “computing methodologies”[MeSH] or “video recording”[MeSH] OR technology[tiab] or robot*[tiab] or technologies[tiab] or “smart home”[tiab] or “smart homes”[tiab] OR computer*[tiab] OR multimedia[tiab] or tablet[tiab] or tablets[tiab] or ipad[tiab] or ipads[tiab] OR video[tiab] or videos[tiab] or videorecording[tiab] or videorecordings[tiab] OR “virtual reality”[tiab] OR “artificial intelligence”[tiab] | 1659120 |
| 2 | rehabilitation[MeSH] OR rehabilitation[sh] or rehabilitat*[tiab] or “daily living”[tiab] OR "daily activities"[tiab] OR "daily activity"[tiab] OR "cognitive training"[tiab] | 480741 |
| 3 | “cognitive dysfunction”[MeSH] or “mild cognitive”[tiab] or “mild neurocognitive”[tiab] | 15486 |
| 4 | #1 AND #2 AND #3 | 229 |
| 5 | #4 (randomized controlled trial[pt] OR controlled clinical trial[pt] OR randomized[tiab] OR randomised[tiab] OR randomization[tiab] OR randomisation[tiab] OR placebo[tiab] OR randomly[tiab] OR trial[tiab] OR groups[tiab] OR Clinical trial[pt] OR “clinical trial”[tiab] OR “clinical trials”[tiab] OR "evaluation studies"[Publication Type] OR "evaluation studies as topic"[MeSH Terms] OR "evaluation study"[tiab] OR evaluation studies[tiab] OR "intervention study"[tiab] OR "intervention studies"[tiab]) NOT (Editorial[ptyp] OR Letter[ptyp] OR Case Reports[ptyp] OR Comment[ptyp]) NOT (animals[mh] NOT humans[mh]) | 105 |

Database: CINAHL Complete

| Set # |  | Results |
| --- | --- | --- |
| 1 | MH "Technology+" OR MH "Computers and Computerization+" OR MH "Videorecording" OR TI (technology or robot* or technologies or “smart home” or “smart homes” OR computer* OR multimedia or tablet OR tablets or ipad OR ipads OR video or videos or videorecording or videorecordings OR “virtual reality” OR “artificial intelligence”) OR AB (technology or robot* or technologies or “smart home” or “smart homes” OR computer* OR multimedia or tablet OR tablets or ipad OR ipads OR video or videos or videorecording or videorecordings OR “virtual reality” OR “artificial intelligence”) | 702734 |
| 2 | MH "Rehabilitation+" OR MJ "rehabilitation" OR TI (rehabilitat* or “daily living” OR "daily activities" OR "daily activity" OR "cognitive training") OR AB (rehabilitat* or “daily living” OR "daily activities" OR "daily activity" OR "cognitive training") | 288625 |
| 3 | TI (“mild cognitive” or “mild neurocognitive”) OR AB (“mild cognitive” or “mild neurocognitive”) | 4923 |
| 4 | #1 AND #2 AND #3 | 175 |
| 5 | #4 AND (ZT "randomized controlled trial" OR MH "Randomized Controlled Trials" OR TI ("randomized controlled trial" OR "controlled clinical trial" OR "randomized" OR "randomized" OR "randomization" OR "randomization" OR "placebo" OR "randomly" OR "trial" OR "groups" OR "evaluation study" OR "evaluation studies" OR "intervention study" OR "intervention studies") OR AB ("randomized controlled trial" OR "controlled clinical trial" OR "randomized" OR "randomized" OR "randomization" OR "randomization" OR "placebo" OR "randomly" OR "trial" OR "groups" OR "evaluation study" OR "evaluation studies" OR "intervention study" OR "intervention studies")) NOT PT (Commentary OR Editorial OR Letter) | 74 |

Database: PsycINFO

| Set # |  | Results |
| --- | --- | --- |
| 1 | DE "Technology" OR DE "Assistive Technology" OR DE "Biotechnology" OR DE "Engineering" OR DE "Information Technology" OR DE "Nanotechnology" OR DE "Nuclear Technology" OR DE "Technology Transfer" OR DE "Computers" OR DE "Artificial Intelligence" OR DE "Artificial Neural Networks" OR DE "Expert Systems" OR DE "Knowledge Engineering" OR DE "Machine Learning" OR DE "Automation" OR DE "Computer Applications" OR DE "Artificial Intelligence" OR DE "Cloud Computing" OR DE "Computer Assisted Design" OR DE "Computer Assisted Diagnosis" OR DE "Computer Assisted Instruction" OR DE "Computer Assisted Testing" OR DE "Computer Assisted Therapy" OR DE "Computer Simulation" OR DE "Electronic Learning" OR DE "Groupware" OR DE "Hypermedia" OR DE "Hypertext" OR DE "Computer Assisted Therapy" OR DE "Computer Peripheral Devices" OR DE "Video Display Units" OR DE "Computer Software" OR DE "Decision Support Systems" OR DE "Groupware" OR DE "Word Processing" OR DE "Human Computer Interaction" OR DE "Internet Usage" OR DE "Mobile Devices" OR DE "Cellular Phones" OR DE "Robotics" OR DE "Videotapes" OR TI (technology or robot* or technologies or “smart home” or “smart homes” OR computer* OR multimedia or tablet OR tablets or ipad OR ipads OR video or videos or videorecording or videorecordings OR “virtual reality” OR “artificial intelligence”) OR AB (technology or robot* or technologies or “smart home” or “smart homes” OR computer* OR multimedia or tablet OR tablets or ipad OR ipads OR video or videos or videorecording or videorecordings OR “virtual reality” OR “artificial intelligence”) | 248108 |
| 2 | DE "Activities of Daily Living" OR DE "Geriatric Assessment" OR DE "Rehabilitation" OR DE "Cognitive Rehabilitation" OR DE "Criminal Rehabilitation" OR DE "Drug Rehabilitation" OR DE "Neuropsychological Rehabilitation" OR DE "Neurorehabilitation" OR DE "Occupational Therapy" OR DE "Physical Therapy" OR DE "Psychosocial Rehabilitation" OR MJ "rehabilitation" OR TI (rehabilitat* or “daily living” OR "daily activities" OR "daily activity" OR "cognitive training") OR AB (rehabilitat* or “daily living” OR "daily activities" OR "daily activity" OR "cognitive training") | 122344 |
| 3 | TI (“mild cognitive” or “mild neurocognitive”) OR AB (“mild cognitive” or “mild neurocognitive”) | 8595 |
| 4 | #1 AND #2 AND #3 | 113 |
| 5 | #4 AND (ZC "treatment outcome/clinical trial" OR DE "Clinical Trials" OR TI ("randomized controlled trial" OR "controlled clinical trial" OR "randomized" OR "randomized" OR "randomization" OR "randomization" OR "placebo" OR "randomly" OR "trial" OR "groups" OR "evaluation study" OR "evaluation studies" OR "intervention study" OR "intervention studies") OR AB ("randomized controlled trial" OR "controlled clinical trial" OR "randomized" OR "randomized" OR "randomization" OR "randomization" OR "placebo" OR "randomly" OR "trial" OR "groups" OR "evaluation study" OR "evaluation studies" OR "intervention study" OR "intervention studies")) NOT (Column/Opinion OR Editorial OR Letter) | 48 |

Database: Embase

| Set # |  | Results |
| --- | --- | --- |
| 1 | 'technology'/exp OR 'computer analysis'/exp OR 'videorecording'/exp OR technology:ab,ti or robot*:ab,ti or technologies:ab,ti or 'smart home':ab,ti or 'smart homes':ab,ti OR computer*:ab,ti OR multimedia:ab,ti or tablet:ab,ti or tablets:ab,ti or ipad:ab,ti OR ipads:ab,ti OR video:ab,ti or videos:ab,ti or videorecording:ab,ti or videorecordings:ab,ti OR 'virtual reality':ab,ti OR 'artificial intelligence':ab,ti | 1116332 |
| 2 | 'rehabilitation'/exp OR 'rehabilitation':lnk OR rehabilitat*:ab,ti or “daily living”:ab,ti OR "daily activities":ab,ti OR "daily activity":ab,ti OR "cognitive training":ab,ti | 565502 |
| 3 | 'mild cognitive impairment'/exp OR “mild cognitive”:ab,ti or “mild neurocognitive”:ab,ti | 24040 |
| 4 | #1 AND #2 AND #3 | 193 |
| 5 | #4 NOT ('case report'/exp OR 'case study'/exp OR 'editorial'/exp OR 'letter'/exp OR 'note'/exp) | 179 |
| 6 | #5 AND [humans]/lim AND [embase]/lim NOT [medline]/lim | 93 |

Database: Cochrane Library

| Set # |  | Results |
| --- | --- | --- |
| 1 | [mh "technology"] OR [mh “computing methodologies”] or [mh “video recording”] OR technology:ab,ti or robot*:ab,ti or technologies:ab,ti or 'smart home':ab,ti or 'smart homes':ab,ti OR computer*:ab,ti OR multimedia:ab,ti or tablet:ab,ti or tablets:ab,ti or ipad:ab,ti OR ipads:ab,ti OR video:ab,ti or videos:ab,ti or videorecording:ab,ti or videorecordings:ab,ti OR 'virtual reality':ab,ti OR 'artificial intelligence':ab,ti | 69377 |
| 2 | [mh rehabilitation] OR rehabilitat*:ab,ti or “daily living”:ab,ti OR "daily activities":ab,ti OR "daily activity":ab,ti OR "cognitive training":ab,ti | 48729 |
| 3 | [mh “cognitive dysfunction”] OR “mild cognitive”:ab,ti or “mild neurocognitive”:ab,ti | 1589 |
| 4 | #1 AND #2 AND #3 | 92 |
| 5 | Limit to Trials | 90 |

**Table S2. Results of quality assessment based on JBI critical appraisal checklist for randomized controlled trails***

| **First Author, year** | **Design** | **C1** | **C2** | **C3** | **C4** | **C5** | **C6** | **C7** | **C8** | **C9** | **C10** | **C11** | **C12** | **C13** |
| --- | --- | --- | --- | --- | --- | --- | --- | --- | --- | --- | --- | --- | --- | --- |
| Hughes, 2014 | RCT | 3 | 3 | 1 | 3 | 3 | 3 | 1 | 1 | 1 | 1 | 1 | 1 | 1 |
| Barban, 2016 | RCT | 3 | 3 | 1 | 3 | 3 | 3 | 1 | 1 | 1 | 1 | 1 | 1 | 1 |
| Heyer, 2016 | RCT | 3 | 3 | 1 | 1 | 1 | 3 | 1 | 1 | 1 | 1 | 1 | 1 | 1 |
| Herrera, 2012 | RCT | 3 | 3 | 3 | 3 | 3 | 3 | 1 | 1 | 1 | 1 | 1 | 1 | 1 |
| Finn, 2011 | RCT | 1 | 3 | 1 | 3 | 3 | 3 | 1 | 1 | 1 | 1 | 1 | 1 | 1 |
| Fiatarone Singh, 2014 | RCT | 1 | 1 | 1 | 1 | 1 | 3 | 1 | 1 | 1 | 1 | 1 | 1 | 1 |
| Delbroek, 2017 | RCT | 3 | 3 | 1 | 3 | 3 | 3 | 1 | 1 | 1 | 1 | 1 | 1 | 1 |
| Rozzini, 2007 | RCT | 3 | 3 | 3 | 3 | 3 | 3 | 1 | 1 | 1 | 1 | 1 | 1 | 1 |
| Gagnon, 2012 | RCT | 3 | 3 | 1 | 3 | 3 | 3 | 1 | 1 | 1 | 1 | 1 | 1 | 1 |
| Bahar-Fuchs, 2017 | RCT | 1 | 1 | 1 | 1 | 1 | 1 | 1 | 1 | 1 | 1 | 1 | 1 | 1 |
| Barnes. 2009 | RCT | 3 | 1 | 1 | 1 | 3 | 3 | 1 | 1 | 1 | 1 | 1 | 1 | 1 |
| Hagovská, 2017 | RCT | 1 | 1 | 1 | 3 | 3 | 3 | 1 | 1 | 1 | 1 | 1 | 1 | 1 |
| Lin, 2016 | RCT | 3 | 1 | 1 | 1 | 3 | 3 | 1 | 1 | 1 | 1 | 1 | 1 | 1 |
| Rosen, 2011 | RCT | 3 | 1 | 1 | 1 | 3 | 3 | 1 | 1 | 1 | 1 | 1 | 1 | 1 |
| Savulich, 2017 | RCT | 3 | 3 | 1 | 3 | 3 | 3 | 1 | 1 | 1 | 1 | 1 | 1 | 1 |

* 1= Yes, 2= No, 3= Unclear, 4= Not applicable

**Table S3. Results of quality assessment based on JBI critical appraisal checklist for quasi-experimental studies***

| **First Author, year** | **Design** | **C1** | **C2** | **C3** | **C4** | **C5** | **C6** | **C7** | **C8** | **C9** |
| --- | --- | --- | --- | --- | --- | --- | --- | --- | --- | --- |
| Cipriani, 2006 | Pre-post study | 1 | 1 | 1 | 2 | 1 | 1 | 1 | 1 | 1 |
| Han, 2014 | Pre-post study | 3 | 2 | 3 | 2 | 1 | 1 | 1 | 1 | 1 |
| Vermei, 2016 | Pre-post study | 3 | 2 | 3 | 1 | 1 | 1 | 1 | 1 | 1 |
| Styliadis, 2015 | CCT | 1 | 1 | 1 | 1 | 1 | 1 | 1 | 1 | 1 |
| Manera, 2015 | Pre-post study | 3 | 2 | 3 | 2 | 1 | 1 | 1 | 1 | 1 |
| Gooding, 2016 | CCT | 1 | 1 | 1 | 1 | 1 | 1 | 1 | 1 | 1 |
| Gonzalez-Palau, 2014 | Pre-post study | 3 | 2 | 3 | 2 | 1 | 1 | 1 | 1 | 1 |
| Talassi, 2007 | CCT | 1 | 1 | 1 | 1 | 3 | 1 | 1 | 1 | 1 |
| Man, 2012 | CCT | 1 | 1 | 1 | 1 | 1 | 1 | 1 | 1 | 1 |
| Klados, 2016 | CCT | 1 | 1 | 1 | 1 | 1 | 1 | 1 | 1 | 1 |
| Mansbach, 2017 | CCT | 1 | 1 | 1 | 1 | 1 | 1 | 1 | 1 | 1 |

* 1= Yes, 2= No, 3= Unclear, 4= Not applicable

**Figure S1**: Forest plot - RBANS score (computer-based intervention VS. Control)


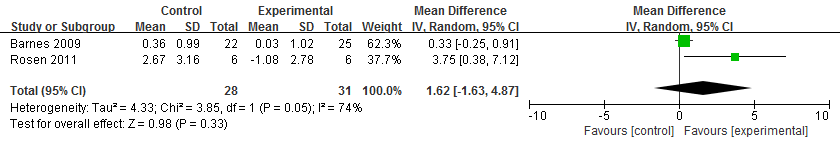

Supplement: Supplementary file 1 — Table S1. Searching Strategy. Table S2. Results of quality assessment based on JBI critical appraisal checklist for randomized controlled trials*. Table S3. Results of quality assessment based on JBI critical appraisal checklist for quasi-experimental studies*. Figure S1. Forest plot - RBANS score (computer-based intervention VS. Control). (DOCX 34 kb) [file 12877_2018_893_MOESM1_ESM.docx]
